# The Contribution of Natural Killer Complex Loci to the Development of Experimental Cerebral Malaria

**DOI:** 10.1371/journal.pone.0093268

**Published:** 2014-04-01

**Authors:** Diana S. Hansen, Victoria Ryg-Cornejo, Lisa J. Ioannidis, Chris Y. Chiu, Ann Ly, Catherine Q. Nie, Anthony A. Scalzo, Louis Schofield

**Affiliations:** 1 The Walter and Eliza Hall Institute of Medical Research, Parkville, Victoria, Australia; 2 Burnet Institute, Melbourne, Victoria, Australia; 3 Department of Medical Biology, The University of Melbourne, Parkville, Victoria, Australia; 4 Centre for Experimental Immunology, Lions Eye Institute, Nedlands, Western Australia, Australia; 5 Centre for Ophthalmology and Visual Science, The University of Western Australia, Nedlands, Western Australia, Australia; 6 Australian Institute of Tropical Health and Medicine, James Cook University, Douglas, Queensland, Australia; University of Sydney, Australia

## Abstract

**Background:**

The Natural Killer Complex (NKC) is a genetic region of highly linked genes encoding several receptors involved in the control of NK cell function. The NKC is highly polymorphic and allelic variability of various NKC loci has been demonstrated in inbred mice, providing evidence for NKC haplotypes. Using BALB.B6-Cmv1^r^ congenic mice, in which NKC genes from C57BL/6 mice were introduced into the BALB/c background, we have previously shown that the NKC is a genetic determinant of malarial pathogenesis. C57BL/6 alleles are associated with increased disease-susceptibility as BALB.B6-Cmv1^r^ congenic mice had increased cerebral pathology and death rates during *P. berghei* ANKA infection than cerebral malaria-resistant BALB/c controls.

**Methods:**

To investigate which regions of the NKC are involved in susceptibility to experimental cerebral malaria (ECM), intra-NKC congenic mice generated by backcrossing recombinant F2 progeny from a (BALB/c x BALB.B6-Cmv1^r^) F1 intercross to BALB/c mice were infected with *P. berghei* ANKA.

**Results:**

Our results revealed that C57BL/6 alleles at two locations in the NKC contribute to the development of ECM. The increased severity to severe disease in intra-NKC congenic mice was not associated with higher parasite burdens but correlated with a significantly enhanced systemic IFN-γ response to infection and an increased recruitment of CD8^+^ T cells to the brain of infected animals.

**Conclusions:**

Polymorphisms within the NKC modulate malarial pathogenesis and acquired immune responses to infection.

## Introduction

Malaria is one the most serious infectious diseases of humans with ∼250 million clinical cases annually. Most cases of severe disease are caused by the blood stage of *Plasmodium falciparum*. [1]. Fatalities are associated with various disease syndromes including respiratory distress, metabolic acidosis, hypoglycaemia, renal failure, pulmonary oedema and cerebral malaria (CM) [2]. This disease syndrome, which accounts for nearly 1 million deaths every year [3] is characterised by seizures and coma. To avoid clearance in the spleen, mature forms of blood-stage malaria adhere to vascular endothelial cells. This process, known as parasite sequestration is thought to induce obstructions in blood flow resulting in hypoxia and haemorrhages [1] that are associated with the development of organ-specific syndromes such CM. Disease-association studies indicate that in addition to parasite sequestration, inflammatory responses also contribute to severe disease [4]. High TNF, IFN-γ and IL-1β levels have been associated with disease severity [4]. Inflammatory chemokines including MIP-1α, MIP-1β [5] and CXCL10 [6], [7] have been found to be associated with increased risk of CM mortality. Although parasite sequestration is the most common feature of patients succumbing to CM, post-mortem examination has also revealed intra and peri-vascular pathology including the presence of leukocytes within brain blood vessels [8]–[11]. These findings suggested that intravascular infiltration of host leukocytes might also contribute to the pathogenesis of some CM cases.

Much useful evidence on the inflammatory processes contributing to the induction of CM has been provided by the *Plasmodium berghei* ANKA model [12]. Like in humans, *P. berghei* ANKA parasitised red blood cells (pRBCs) have been found to bind to brain vascular endothelial cells [13]. Inflammatory responses mediated by the cytokines TNF [14] and IFN-γ [15] and the chemokine CXCL10 [16] have been shown to contribute to severe malaria in mice. Several leukocyte populations including macrophages, neutrophils, T cells, NK cells and platelets have been found in brain blood vessels of CM-affected mice during infection [17]–[21]. From those populations, CD8^+^ T cells are highly abundant and were shown to mediate CM in a perforin-dependent manner [17], [21].

Elements of the innate immune system have also been shown to contribute to the development of ECM [22]. CD1d-restricted NKT cells have been shown to play a protective role against *P. berghei* ANKA-mediated CM in BALB/c mice but induce early IFN-γ production and promote disease in C57BL/6 susceptible animals [23]. Like NKT cells, NK cells readily secrete IFN-γ in response to malaria in humans [24], [25] and mice [26]. Furthermore, NK cells were shown to facilitate the recruitment of CXCR3^+^ T cells to the brain of malaria-infected mice in an IFN-γ-mediated manner [19]. More specifically, NK cells stimulate the dendritic cell (DC)-mediated priming of CD8^+^ T cells in response to *P. berghei* ANKA [27].

NK and NKT cell function is controlled by surface receptors encoded within a genetic region called NKC [28]. Many of these genes encode type II integral membrane proteins that have inhibitory or activation function depending on the presence or absence of immunoreceptor tyrosine-based inhibitory motifs (ITIMs) in their intracellular domains. Upon ligation, ITIMs become phosphorylated and recruit protein tyrosine phosphatases, which interferes with cell activation. In the mouse, inhibitory receptors include the Ly49 superfamily and NKG2 molecules, expressed as heterodimers with CD94. Ly49 receptors interact with MHC I molecules, and CD94/NKG2 complexes bind to Qa-1^b^, a non-classical MHC I receptor. Interaction of these receptors with their MHC I ligands induces inhibition of cytotoxic activity by NK cells. Under pathological conditions such as viral infections or tumors, the expression of MHC I molecules is down-regulated, resulting in loss of negative regulation by inhibitory receptors, NK cell activation and killing of target cells [29]. Some members of the Ly49 family lack ITIMs and have activation function. Stimulation of activation receptors such as Ly49D [30] and Ly49H [31] leads to IFN-γ secretion and cytotoxic activity. Other activation receptors encoded within the murine NKC are CD94/NKG2C, NKG2D and NK1.1. In humans, NK receptors are encoded by 2 regions: the NKC on chromosome 12 and the killer cell Ig-like receptor (KIR) region on chromosome 19. Control of NK cell function in humans is mediated by interactions between HLA I and KIR molecules. Thus mouse Ly49 and human KIR genes are functional homologues, which illustrates an intriguing example of convergent evolution [32].

The NKC on mouse chromosome 6 is a polymorphic region. Allelic variability of loci has been shown in inbred mice providing evidence for NKC haplotypes [33]. In malaria, C57BL/6 NKC alleles are associated with disease susceptibility, as congenic BALB.B6-Cmv1^r^, in which NKC genes from C57BL/6 mice were introduced in the BALB/c background showed increased cerebral pathology, pulmonary oedema, anaemia and death rates during *P. berghei* ANKA infection [34], compared to resistant BALB/c controls. To date, the specific NKC receptors involved in the induction of malarial pathogenesis have not been identified. To address this question in this study intra-NKC recombinant mouse strains bearing small intervals of the C57BL/6 NKC in the BABL/c background were infected with *P. berghei*-ANKA. Our results revealed that alleles at two locations in the NKC contribute to susceptibility to CM in this model.

## Methods

### Ethics Statement

This manuscript contains work carried out with experimental mice. In *P. berghei* ANKA-infected mice, clinical illness develops between days 6–10 p.i. During this period, *P. berghei*-infected mice were monitored 5 times at 8:00 AM, 11:00 AM, 3:00 PM and 6:00 PM and 10:00PM. Mice developing loss of self-righting reflex were humanely euthanized by CO_2_ inhalation or cervical dislocation. All experiments were performed in compliance with the Walter & Eliza Hall Institute Animal Ethics Committee requirements. The Walter & Eliza Hall Institute Animal Ethics Committee has approved this study.

### Mice and infections

Eight to 12-week old BALB/c, C567BL/6, BALB.B6-Cmv1^r^, BALB.B6-CT-1, BALB.B6-CT-6, BALB.B6-CT1-2 and BALB.B6-CT-13 [35] were used throughout the study. All mice were bred in The Walter & Eliza Hall Institute animal facility. Groups of 10–20 mice were injected intraperitoneally (i.p.) with 1×10^6^ freshly passaged *P. berghei*-ANKA pRBC. Parasitemia was assessed by counting 10 microscope fields from Giemsa-stained smears of tail blood prepared every 2–3 days. Mortality was checked daily. Mice were judged as developing CM if they displayed neurological signs such as ataxia, loss of reflex and hemiplegia.

### *In Vivo* bioluminescence imaging

Mice were infected (1×10^5^ pRBC, i.v.) with a transgenic *P. berghei*-ANKA line expressing luciferase and GFP under the control of the elongation factor 1-α promoter [36]. Luciferase-expressing pRBCs were visualized in the brain with an I-CCD photon-counting video camera and *in vivo* imaging system (IVIS 100; Xenogen, Alameda, CA). Bioluminescence generated by luciferase transgenic parasites in brain tissue was measured according to the manufacturer's instructions using the same regions of measurement for all samples being compared.

### Flow cytometry

Spleen cells from BALB/c, C57BL/6, BALB.B6-CT-1, BALB.B6-CT-6, BALB.B6-CT-12 and BALB.B6-CT-13 mice were incubated with anti-CD16 antibody (Fc-block), washed and then stained with PE-conjugated anti-CD49b (DX-5) and PE-CY5-conjugated anti-TCR (H57-597) antibodies. Some samples were simultaneously stained with other antibodies such as FITC-conjugated anti-Ly49A (A1), Ly49D (4E5), Ly49G_2_ (Cwy-e), Ly49I (YLI-90), anti-NKG2A/C/E (20d5) or anti-NK1.1 (PK136) for 1 h on ice (all antibodies and conjugates are from BD Pharmingen, San Diego, CA, USA). The cells were then washed twice, resuspended in PBS and analysed in a FACScalibur cytofluorometer (BD Biosciences, NJ) using CellQuest software. Viable lymphocytes were gated by forward and side scatter.

### ELISA for IFN-γ detection

Ninety-six-well plates were coated with capture antibody (R4-6A2) by overnight incubation at 4°C in Phosphate Buffer pH 9. Plates were then blocked with 1% BSA for 1 h at 37°C. Serum samples were diluted 1/5 and tested in triplicates by overnight incubation at 4°C. Plates were then incubated for 2 h at 20°C with the biotinylated antibody (XMG1-2) and then for 1 h with streptavidin-peroxidase conjugate (Pierce, Rockford, IL). Bound complexes were detected with tetramethyl-benzidine (KBL, Gaithersburg, MD) and H_2_O_2_. Absorbance was read at 450 nm. Cytokine concentration was calculated using recombinant IFN-γ for the preparation of standard curves.

### Purification and analysis of brain-sequestered leukocytes

Brain-sequestered leukocytes were purified on day 7 p.i with *P. berghei*-ANKA as described before [19]. Briefly, euthanized mice were perfused to remove circulating leukocytes. Brains were then removed, crushed in RPMI medium and pushed through a cell mesh. The tissue extract was centrifuged at 200× g for 10 min and the pellet was dissolved in RPMI containing 0.05% Collagenase D (Worthington, Lakewood, NJ) and 2 U/ml DNAase I (Sigma). After 1 h incubation at 22°C, the mixture was filtered through a cell strainer, seeded on a 35% Percoll (Amersham Bioscience, Uppsala, Sweden) cushion and centrifuged at 400× g for 20 min at 22°C. The pellet was collected and erythrocytes were lysed with Tris-NH_4_Cl Buffer. After washing, recovered cells were incubated with anti-CD16 antibody, washed and stained with PE-anti-NK1.1 (PK136), FITC-anti-TCR (H57-597) and PerCP-Cy5.5-anti-CD8 (53-6.7). After washing, cells were resuspended in PBS and analysed by flow cytometry.

### Statistical analysis

A one-way ANOVA was used for data evaluation. A Tukey's multiple comparison post-test was used to evaluate differences between individual mouse strains. Differences in mortality rates of *P. berghei* infected mice were assessed by Cox-Mantel logrank analysis.

## Results

### Differential expression of markers NKC in intra-NKC congenic mice

**Figure 1:**
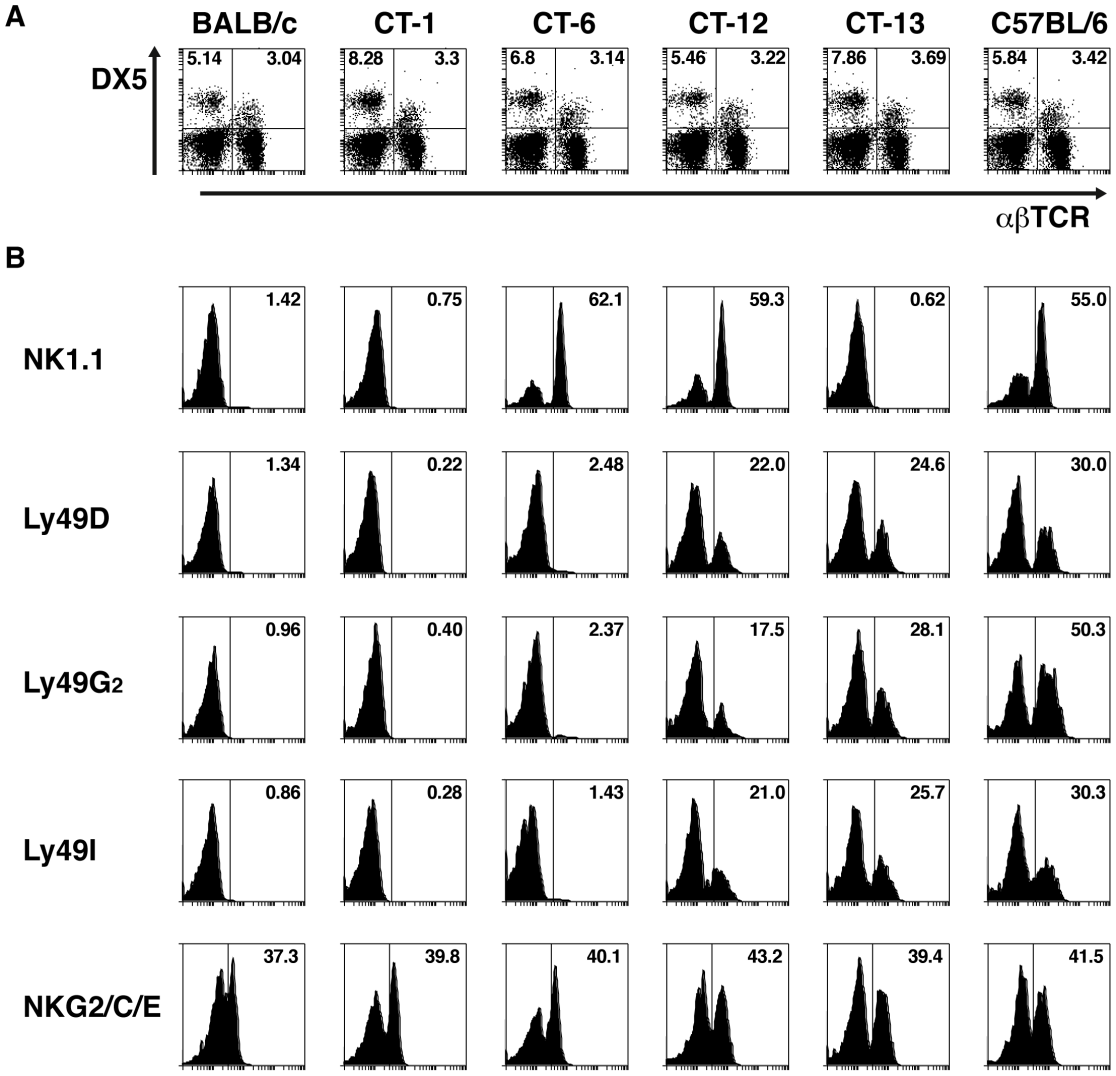
Differential expression of NKC markers in NK/NKT cells from BALB.B6-CT-1, BALB.B6-CT-6, BALB.B6-CT-12 and BALB.B6-CT-13 mice. (*A*) Spleen cells from C57BL6, BALB/c and BALB.B6-CT-1, BALB.B6-CT-6, BALB.B6-CT-12 and BALB.B6-CT-13 mice were stained with anti-CD49b (DX5) and anti-αβ TCR antibodies. The percentage of NK and NKT cells are indicated. (*B*) The expression of the NKC markers NK1.1, Ly49A, Ly49D, Ly49G_2_, Ly49I, and NKG2A/C/E was calculated on all DX5 positive cells from the 6 mouse strains. Representative histograms are shown.

BALB.B6-Cmv1^r^ mice are a congenic strain in which the NKC from C57BL6 mice has been introduced into the BALB/c background [37]. As the NKC encodes several different receptors, intra-NKC recombinant mouse strains were generated by backcrossing recombinant F2 progeny from a (BALB/c x BALB.B6-Cmv1^r^) F1 intercross to BALB/c mice [35]. These congenic strains are a valuable tool to facilitate mapping of phenotypically-defined loci [35]. The genotypes of congenic strains used in this study are described in Table 1. To determine the phenotypic properties of NK and NKT cells from wild-type and congenic mice, spleen cells from BALB/c, C57BL/6 BALB.B6-CT-1, BALB.B6-CT-6, BALB.B6-CT-12 and BALB.B6-CT-13 mice were stained with antibodies against the pan NK-NKT cell marker DX-5 and αβTCR. Figure 1A shows that spleens of all mouse strains tested have similar percentages of both NK and NKT cells. The expression of different NKC markers was then studied in all DX5 positive splenocytes from the 6 mouse strains. Antibodies directed to C57BL/6 NKC receptors such as NK1.1, Ly49D, Ly49G_2_ and Ly49I recognized these molecules on the surface of cells from wild-type C57BL/6 as well as BALB.B6-CT-12 mice (Figure 1B). None of these molecules could be detected in spleen cells from BALB/c wild-type or BALB.B6-CT-1 mice, which only express C57BL/6 alleles outside the NKC (Table 1). Recombinations in BALB.B6-CT-6 and BALB.B6-CT-13 mice divide the NKC interval expressed by the as BALB.B6-CT-12 strain in 2 regions (Table 1). DX5^+^ cells from BALB.B6-CT-6 mice expressed NK1.1 but were unable to be recognised by antibodies against members of the Ly49 superfamily. In contrast, cells from BALB.B6-CT-13 mice readily expressed Ly49D, Ly49G_2_ and Ly49I but did not react with an anti-NK1.1 antibody. Antibodies directed against NKG2A/C/E were able to detect these molecules on all mouse strains tested, suggesting a higher homology level (Figure 1B). Thus NK and NKT cells from intra-NKC congenic mice differ in the expression of NKC markers such as NK1.1 and members of the Ly49 gene superfamily.

**Table 1:** Genotypes of intra-NKC congenic strains.

	Cmv1^r^	CT-1	CT-6	CT-12	CT-13
*D6Mit108*	c	c	c	c	c
*Tnfr1*	**b**	c	**b**	**b**	c
*D6Mit52*	**b**	c	**b**	**b**	c
*Nkrp1a*	**b**	c	**b**	**b**	c
*D6Mit135*	**b**	c	**b**	**b**	c
*Cd69*	**b**	c	**b**	**b**	c
*CD94*	**b**	c	c	**b**	**b**
*Ly49a*	**b**	c	c	**b**	**b**
*Cmv1 (Ly49h)*	**b**	c	c	**b**	**b**
*D6Mit13*	**b**	**b**	c	c	c
*D6Mit25*	**b**	**b**	c	c	c
*D6Mit59*	c	c	c	c	c

c: BALB/c allele, **b**: C57BL/6 allele.

### C57BL/6 alleles at two locations in the NKC contribute to susceptibility to ECM

**Figure 2:**
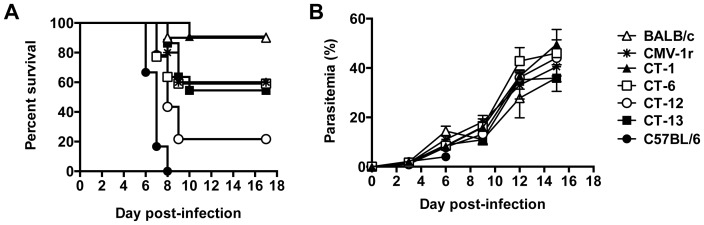
Control of malarial pathogenesis by the NKC. Groups of BALB/c, C567BL/6, BALB.B6-*Cmv1^r^*, BALB.B6-CT-1, BALB.B6-CT-6, BALB.B6-CT1-2 and BALB.B6-CT-13 mice (n = 10–20) were infected with *P. berghei* ANKA. (*A*) The percentage survival was monitored daily. (*B*) Parasitemia was assessed from Giemsa-stained blood smears. Each point represents the mean of 6–10 samples ± SD.

We have previously shown that BALB.B6-Cmv1^r^ congenic mice display partial susceptibility to *P. berghei* ANKA-mediated severe malaria [34]. To investigate which regions of the NKC are involved in susceptibility to CM, intra-congenic mouse strains were infected with *P. berghei*-ANKA and survival monitored daily. Consistent with previous observations, BALB.B6-Cmv1^r^ mice developed CM symptoms and 40% of the animals succumbed to diseased by day 9 post-infection (p.i.). The strain BALB.B6-CT-12, which bears most of the C57BL/6 NKC alleles but lacks a more distal region (defined by the microsatellite markers *D6Mit13*, *D6Mit25* and *D6Mit59*) present in the original BALB.B6-Cmv1^r^ congenic (Table 1), showed significant increased susceptibility to disease than both BALB/c (p = 0.0009) and BALB.B6-Cmv1^r^ mice (p = 0.0264), reflected as 78% fatalities (Figure 2A). The increased penetrance of the susceptibility phenotype in BALB.B6-CT-12 compared to BALB.B6-Cmv1^r^ mice suggests that C57BL/6 loci present in that distal region of BALB.B6-Cmv1^r^ mice might contribute to resistance to severe malaria in this model. Similar to BALB/c wild-type controls, BALB.B6-CT-1 mice that express BALB/c alleles throughout most of the NKC region (Table 1) were highly resistant to *P. berghei* ANKA-mediated CM (Figure 2A). Recombinations in BALB.B6-CT-6 and BALB.B6-CT-13 mice split the NKC interval expressed by the BALB.B6-CT-12 strain in 2 regions (Table 1). Unlike BALB.B6-CT-12 mice, which were nearly as susceptible to disease as C57BL/6 controls, when BALB.B6-CT-6 and BALB.B6-CT-13 animals were challenged with *P. berghei*-ANKA (Figure 2A) they displayed partial susceptibility to ECM (p = 0.0356 between BALB.B6-CT-6 and BALB.B6-CT-12, p = 0.0037 between BALB.B6-CT-13 and BALB.B6-CT-12). Together these results suggest that C57BL/6 alleles at two locations in the NKC contribute to susceptibility to CM in this model.

**Figure 3:**
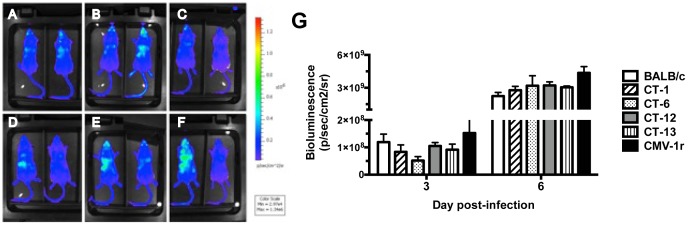
Control of malarial pathogenesis by the NKC does not affect total parasite biomass. (*A*) BALB/c, (*B*) BALB.B6-*Cmv1^r^*, (*C*) BALB.B6-CT-1, (*D*) BALB.B6-CT-6, (*E*) BALB.B6-CT-12 and (*F*) BALB.B6-CT-13 were infected with a luciferase-expressing *P. berghei* ANKA transgenic strain. Whole body parasite biomass acquired on day 3 p.i is shown. Bioluminescence emerging from living parasites was calculated on days 3 and 6 p.i (*G*). Each point represents the mean of 3 samples ± SD. The experiment is representative of 2 separate infection experiments.

Parasite burdens were also evaluated at different time points p.i. Consistent with previous findings with BALB.B6-Cmv1^r^ mice, parasitemia was not affected by the NKC genotype (Figure 2B). As *P. berghei* pRBC accumulate in various target organs, it has been suggested that peripheral parasitemia might not be an accurate estimation of parasite densities. To address this issue, intra-NKC congenic mice were infected with a transgenic *P. berghei*-ANKA line that constitutively expresses luciferase [36]. Following luciferin injection on days 3 and 6 p.i., mice were anesthetised and total parasite biomass was determined by calculating the bioluminescence emerging from living parasites. As expected parasite biomass increased as the infection developed. No significant differences were found in bioluminescence levels detected from parasites in NKC congenic mice (Figure 3 A, B). Thus these results indicate that control of malarial fatalities by the NKC cells does not operate through effects on parasite growth rates.

### The NKC controls systemic IFN-γ in murine severe malaria

**Figure 4:**
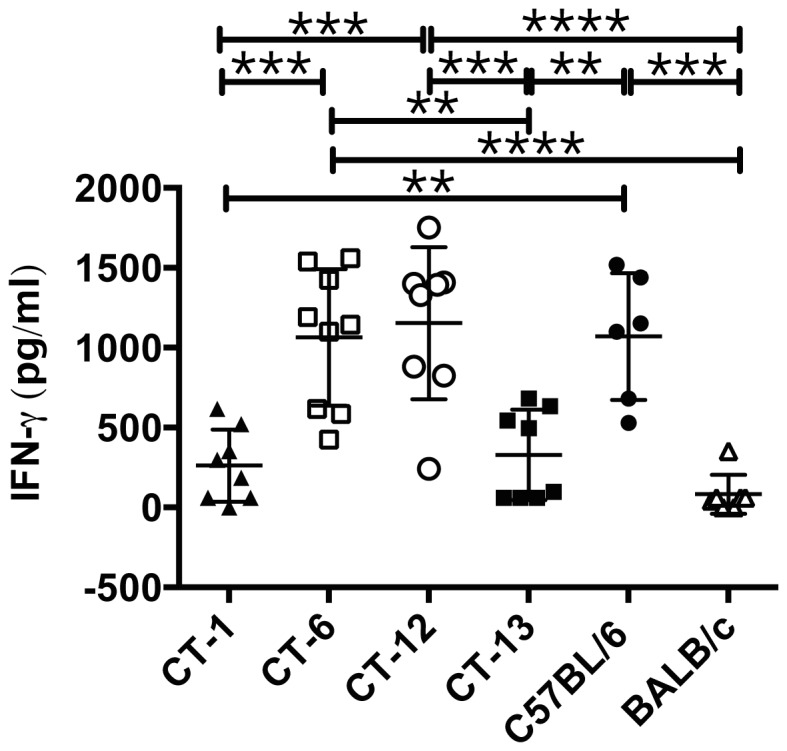
The NKC controls systemic IFN-γ production during malaria infection. Groups of 4–5 BALB/c, C567BL/6, BALB.B6-CT-1, BALB.B6-CT-6, BALB.B6-CT-12 and BALB.B6-CT-13 were infected with *P. berghei* ANKA. IFN-γ levels in sera collected on day 5 p.i. were measured by capture ELISA. The graph shows pooled data from 2 separate infections, which gave similar results. **** P<0.0001, between BALB.B6-CT-12 and BALB/c and between BALB.B6-CT-6 and BALB/c; *** P<0.0005 between BALB.B6-CT-12 and BALB.B6-CT-1, between BALB.B6-CT-12 and BALB.B6-CT-13, between BALB.B6-CT-1 and BALB.B6-CT-6 and between C57BL/6 and BALB/c; ** P<0.005 between BALB.B6-CT-6 and BALB.B6-CT-13, between C57BL/6 and BALB.B6-CT-1 and between C57BL/6 and BALB.B6-CT-13.

IFN-γ is a pro-inflammatory cytokine critical for CM pathogenesis in mice. We have previously found that the NKC differentially regulates systemic IFN-γ levels as well as the transcription of several IFN-γ-inducible genes during ECM [34]. To identify NKC loci responsible for these responses, intra-NKC congenic mouse strains were infected with *P. berghei* ANKA and IFN-γ levels were measured in sera collected on day 5 p.i. by capture ELISA. From all the congenic mouse stains evaluated, BALB.B6-CT-6 and BALB.B6-CT-12 mice secreted IFN-γ levels as high as those produced by C57BL/6 animals (Figure 4). This enhanced IFN-γ response by BALB.B6-CT-6 and BALB.B6-CT-12 mice was significantly higher than that obtained in BALB/c wild-type control mice as well as the CM-resistant BALB.B6-CT-1 congenic strain and BALB.B6-CT-13 animals (Figure 4). Thus together the data suggest that C57BL/6 alleles located within the NKC interval expressed by BALB.B6-CT-6 are required for optimal systemic IFN-γ responses to *P. berghei* ANKA.

### C57BL/6 NKC alleles are required for optimal induction of brain-sequestered CD8^+^ T cells

**Figure 5:**
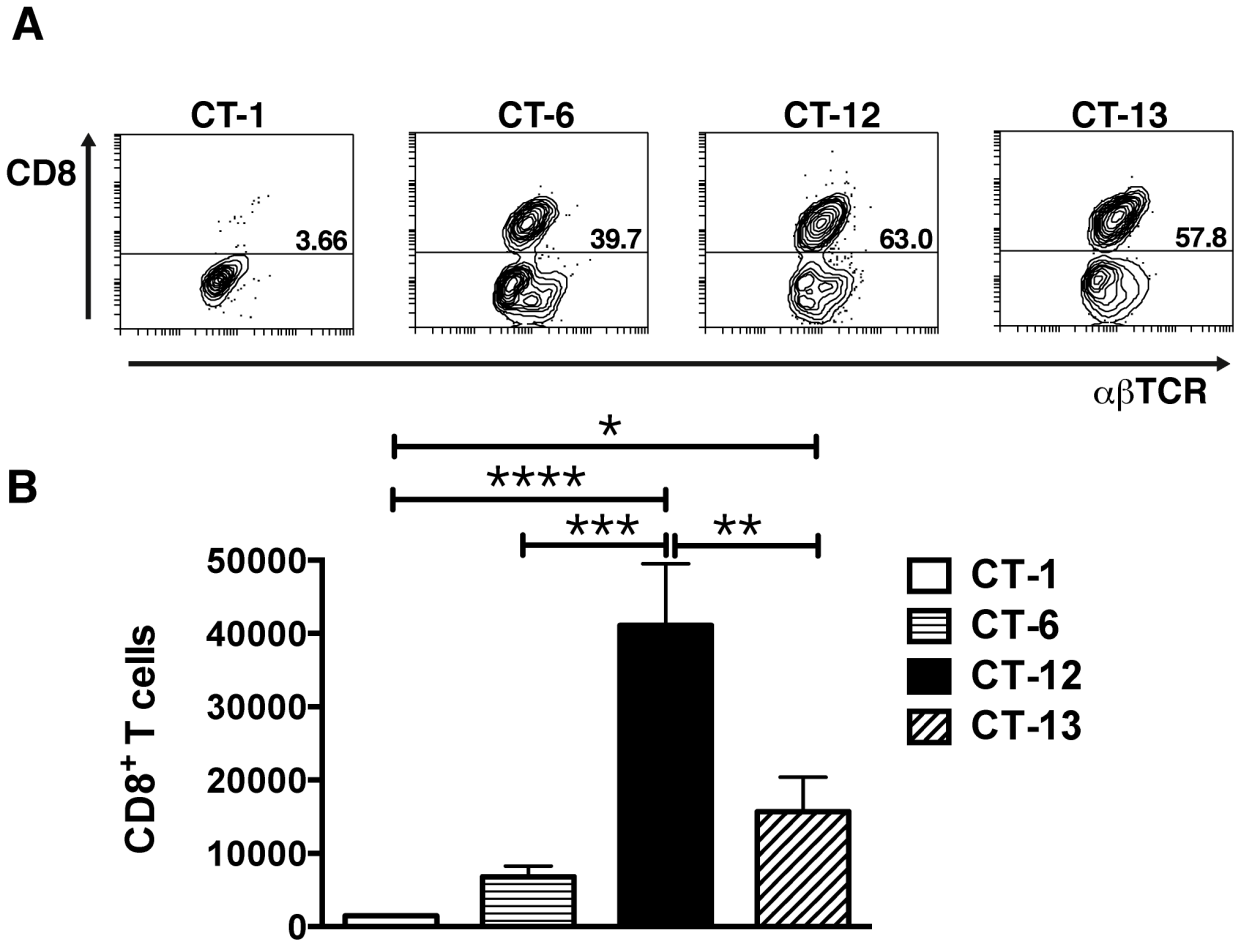
CD8^+^ T cells accumulate in brains of NKC congenic mice during *P. berghei* ANKA infection. BALB.B6-CT-1, BALB.B6-CT-6, BALB.B6-CT-12 and BALB.B6-CT-13 mice were infected with *P. berghei* ANKA. Brains were collected on day 7 p.i after extensive perfusion of the euthanised animals. The BSL were isolated, stained anti-TCR and anti-CD8 antibodies and analysed by flow cytometry. The percentage (*A*) and absolute number (*B*) of CD8^+^ T cells were calculated. Representative dot plots are shown. Each bar represents the mean of 7–8 samples obtained over 2 separate infection experiments that gave similar results ± SEM, **** P<0.0001 between BALB.B6-CT-12 and BALB.B6-CT-1; ***P<0.0005 between BALB.B6-CT-12 and BALB.B6-CT-6; ** P<0.01 between BALB.B6-CT-12 and BALB.B6-CT-13; *P<0.05 between BALB.B6-CT-13 and BALB.B6-CT-1.

The accumulation of CXCR3^+^ CD8^+^T cells in brains of *P. berghei* ANKA infected mice is a key contributing factor in ECM development. Previous work demonstrated that NK cells stimulate the DC-mediated priming of naïve CD8^+^ T cells in response to *P. berghei* ANKA [27] and facilitate the migration of these cells to the brain of infected mice [19]. To determine if different NKC receptors modulate this process, BALB.B6-CT-1, BALB.B6-CT-6, BALB.B6-CT-12 and BALB.B6-CT-13 mice were infected with *P. berghei* ANKA and the percentage and total number of brain-sequestered CD8^+^ T cells was determined on day 7 p.i. Consistent with their increased resistance to ECM, virtually no CD8^+^ T cells could be isolated from brains of BALB.B6-CT-1 mice (Figure 5 A,B). In contrast, 40–60% of total the brain-sequestered T cell pool consisted of CD8^+^ T cells in congenic mice expressing C57BL/6 NKC (Figure 5A). From these mice, the highly CM-susceptible BALB.B6-CT-12 strain had total numbers of brain-sequestered CD8^+^ T cells significantly higher than BALB.B6-CT-1, BALB.B6-CT-6 and BALB.B6-CT-13 mice (Figure 5B). This level of leukocyte sequestration in BALB.B6-CT-12 mice is similar to that previously reported in wild-type C57BL/6 animals [19]. BALB.B6-CT-6 and BALB.B6-CT-13 mice displayed intermediate levels of brain leukocyte sequestration, with absolute numbers of CD8^+^ T cells higher than CM-resistant BALB.B6-CT-1 animals, though only reaching significance for BALB.B6-CT-13 mice. Thus together the data suggest that C57BL/6 alleles in two locations are required for optimal recruitment of CD8^+^ T cells to the brain of *P. berghei* ANKA infected mice.

## Discussion

Previous work demonstrated that the NKC is a significant genetic determinant of murine severe malaria, as congenic BALB.B6-Cmv1^r^ mice showed increased cerebral pathology, pulmonary edema and death rates during *P. berghei* ANKA infection [23] compared to fully resistant BALB/c wild-type controls. In this study infection of intra-NKC congenic mice, generated by backcrossing recombinant F2 progeny from a (BALB/c x BALB.B6-Cmv1^r^) F1 intercross to BALB/c mice [35], revealed that C57BL/6 alleles at two locations in the NKC contribute to the development of ECM. The increased severity to malarial disease in intra-NKC congenic mice was not associated with higher parasite burdens but correlated with a significantly enhanced systemic IFN-γ response and an increased recruitment of CD8^+^ T cells to the brain of infected animals.

A large body of evidence indicates that IFN-γ plays a central role in CM pathogenesis [15], [38]–[43]. *In vitro* studies indicate that human NK cells produce IFN-γ in response to *P. falciparum*-pRBCs [24] and that the differential expression of human NK cell receptors modulates NK cell activation in response to blood stage malaria [44]. Moreover, the differential expression of the NKC-encoded and KIR-encoded receptors NKG2A, CD94, CD158α/KIR2DL1 was found to modulate *P. falciparum*-mediated IFN-γ responses by γδ-T cells [45]. Consistent with those finding, we found that the differential expression of NKC receptors in mice modulates IFN-γ responses to *P. berghei* ANKA and susceptibility to severe disease. Interestingly, although NKC loci at 2 separate locations were found to contribute to the development of ECM, C57BL/6 alleles located within the NKC interval expressed by BALB.B6-CT-6 appeared to be required for optimal systemic IFN-γ responses to infection. Several activation receptors including Ly49D [30], Ly49H [31], NK1.1 [46] and NKG2D [47] have been described to elicit IFN-γ secretion by NK and/or NKT cells upon stimulation. NK1.1 was found to be readily expressed by NK cells and NKT cells of BALB.B6-CT-6 congenic mice, raising the possibility that this receptor might modulate IFN-γ responses to infection. In support of that hypothesis, we have previously shown that cross-linking of NK1.1 preferentially induces IFN-γ and no IL-4 production by CD1d-restricted NKT cells [23], suggesting a TCR-independent pathway of pro-inflammatory responses to infection.

Although initially CD1d-restricted NKT cells appeared to be an important NKC receptor expressing cell lineage responsible for the increase susceptibility to disease in malaria-infected BALB.B6-Cmv1^r^ mice [23], further evidence suggested that IFN-γ production by activated NK cells could also have an impact on the overall immunological status of BALB.B6-Cmv1^r^ mice, influencing not only disease outcome but also innate and adaptive responses to infection [34]. Moreover in fully susceptible C57BL/6 mice, NK cells [19] were found to contribute to the development of ECM by stimulating the recruitment of CD8^+^ T cells to the brain of *P. berghei* ANKA infected mice. Similarly, the present study found using intra-NKC congenic mice (BALB.B6-CT-12) exerting 80% penetrance that NKC loci are required for that process. Thus the highly susceptibility level of congenic BALB.B6-CT-12 mice revealed novel aspects in the control of this response.

IFN-γ has been shown to participate in different responses involved in the development of ECM including, upregulation of receptors mediating parasite sequestration in the vascular endothelium, induction of CXCR3 chemokines responsible for the recruitment of T cells to the brain, etc [16], [48], [49]. Interestingly, although NKC alleles in the interval expressed by BALB.B6-CT-6 were found to be sufficient for the induction of systemic IFN-γ responses, the infection of this congenic strain resulted only in partial recruitment of CD8^+^ T cells to the brain. Similar results were also found after infection of BALB.B6-CT-13 mice that express incomplete C57BL/6 NKC intervals. Together these results are consistent with the notion that at least 2 NKC loci at different locations mediate different non-redundant responses required to fully recapitulate this phenotype. Prior to their CXCR3-mediated recruitment to the brain, the priming of naïve CD8^+^ T cells has been shown to be largely mediated by CD8α^+^ conventional DCs, which appears to be the main subset involved in cross-presentation of parasite-expressed antigens [20]. Interestingly, NK cells and DCs were shown to participate in a cross-talk which results in mutual activation whereby NK cells stimulate IL-12 output by CD8α^+^ conventional DCs required for optimal CD8^+^ T cell priming [19]. Further work is required to determine whether different NKC receptors participate in the induction of these 2 processes (T cell priming and chemokine secretion) required for efficient migration of inflammatory leukocytes to the brain of malaria-infected mice.

The NKC is conserved among species, with syntenic regions identified in rat and human chromosomes. Similar to our findings here, the differential expression of human NK cell receptors modulates NK cell activation in response to *P. falciparum* pRBC [44]. Using a rodent infection model, we established that polymorphisms within NKC loci regulate malarial pathogenesis and the induction of acquired immune responses to infection. Despite the important public health problem that human malaria infections poses worldwide, many immunological and genetic aspects of this disease are not fully understood. Thus further work is required to determine whether NKC receptors are associated with disease severity to malaria in human populations.
